# Estimated prevalence of hepatitis B and C among immigrants in Canada

**DOI:** 10.14745/ccdr.v51i67a01

**Published:** 2025-07-01

**Authors:** Laurence Campeau, Janelle Elliott, Anson Williams, Simone Périnet, Qiuying Yang, Joseph Cox, Jordan J Feld, Christina Greenaway, Nashira Popovic

**Affiliations:** 1 Public Health Agency of Canada, Ottawa, ON; 2 Department of Epidemiology, Biostatistics and Occupational Health, McGill University, Montréal, QC; 3 Toronto Centre for Liver Disease, University Health Network, University of Toronto, Toronto, ON; 4 Division of Infectious Diseases, Jewish General Hospital, McGill University, Montréal, QC

**Keywords:** viral hepatitis, hepatitis B, hepatitis C, prevalence, priority populations, immigrants

## Abstract

**Background:**

Canada’s Sexually Transmitted and Blood-borne Infections (STBBI) Action Plan and the Global Health Sector Strategies on STBBI highlight the importance of putting people at the centre of the health system response. Several key populations are disproportionately affected by viral hepatitis, including immigrants. However, there is a limited body of evidence on the burden of viral hepatitis among immigrants in Canada. We seek to address this gap by estimating the prevalence of hepatitis B (HBV) and C (HCV) infections among immigrants in Canada.

**Methods:**

Using country- and region-specific publicly available data on the prevalence of HBV and HCV, we estimated the number of immigrants with chronic HBV (CHB), HCV antibodies, and chronic HCV (CHC) by multiplying the number of immigrants from Statistics Canada’s 2021 census of population data by the corresponding publicly available country or region-of-origin prevalence, including lower and upper bounds. Each country was categorized as low (<2%) or intermediate-to-high (≥2%) based on published prevalence. To capture changes over time, estimates were stratified by time-period, where possible.

**Results:**

In 2021, the estimated prevalence of viral hepatitis among all immigrants was 4.03% for CHB, 1.43% for HCV antibodies, and 0.78% for CHC. The estimated prevalence of CHB, HCV antibodies, and CHC was 0.91%, 0.96% and 0.52%, respectively, among immigrants from low-prevalence countries (<2%). It was 5.57%, 4.04%, and 2.20%, respectively, among immigrants from intermediate-to-high-prevalence countries (≥2%).

**Conclusion:**

This is the first study to estimate the burden of HBV and HCV among immigrants at the national level in Canada. The results show that the prevalence of viral hepatitis among immigrants is higher than the general Canadian population. However, grouping all immigrants into one category masks important variation, and potentially over-estimates the burden of HBV and HCV among immigrants. Strengthening our understanding of hepatitis prevalence among immigrants can improve our ability to connect those in need to care and treatment services.

## Introduction

Hepatitis B and C are viral infections that pose a significant health threat, as they have the potential to induce chronic liver infection, culminating in severe complications, such as cirrhosis and liver cancer. Recognizing the urgency of this public health challenge, the World Health Organization developed the *Global health sector strategies 2022–2030*, on HIV, viral hepatitis and sexually transmitted infections to guide focused responses by member states towards eliminating sexually transmitted and blood-borne infections (STBBI) by 2030 ((1)). Canada endorsed these global goals and developed the Government of Canada’s STBBI action plan 2024–2030 ((2)), building upon commitments for implementing the pan-Canadian STBBI framework for action ((3)).

These foundational documents ((2,3)) highlight the critical importance of putting people at the centre of the health system response by organizing services around individuals’ needs, rather than around diseases. Several key populations are differentially affected by STBBI, including immigrants. These populations face inequities in accessing care and treatment services for STBBI for a variety of reasons, including stigma and discrimination, language barriers, cultural differences, economic difficulties, and issues related to transportation ((4)). An understanding of the burden of hepatitis B virus (HBV) and hepatitis C virus (HCV) prevalence among all key populations disproportionately impacted by viral hepatitis is needed for public health planning and to support elimination efforts.

In 2021, more than eight million people, or almost one-quarter (23.0%) of the Canadian population, were considered immigrants ((5)), many of whom were born in countries where HBV and HCV is more common. However, there is a limited body of evidence on the burden of viral hepatitis among immigrants in Canada. To our knowledge, only one national-level study from 2006 has examined the prevalence of hepatitis B among immigrants, and no national studies have assessed the prevalence of hepatitis C in this group. This paper seeks to address this gap by estimating the prevalence of HBV and HCV infections among immigrants in Canada, using country-specific epidemiological data.

## Methods

In the context of this study, the Statistics Canada definition of immigrant was used ((6)). An immigrant refers to anyone who has been granted the right to live in Canada permanently by immigration authorities. This includes people who are or who have ever been landed immigrants and permanent residents. It includes those who have obtained Canadian citizenship by naturalization ((6)). Individuals holding work, study or temporary resident permits, as well as those who have claimed refugee status, are considered non-permanent residents, and are therefore excluded from this study.

Data on immigration by country and period of arrival were obtained from Statistics Canada’s 2021 census of population data ((5)). Countries of birth were grouped into world regions according to the regional classification system by Statistics Canada ((5)) for hepatitis B, and the Global Burden of Disease for hepatitis C ((7)).

**Figure 1:**
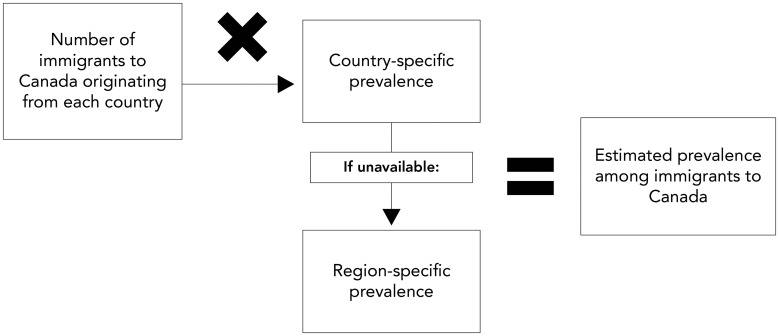
Methodology to estimate hepatitis prevalence among immigrants Note: Statistics Canada time periods of immigration used to estimate chronic HBV (HbsAg): ≤1990, 1991–2000, 2001–2010, 2011–2021; and chronic HCV (HCV RNA): <2016, 2016–2021. Note that time period data not available for anti-HCV Source: Statistics Canada. Immigrant status and period of immigration by place of birth and citizenship: Canada, provinces and territories and census metropolitan areas with parts. Ottawa, ON: StatCan; 2022. https://www150.statcan.gc.ca/t1/tbl1/en/tv.action?pid=9810030201

Chronic hepatitis B (CHB) was defined as HBsAg serology positive. HBsAg seroprevalence estimates were obtained from Wong *et al.* (8). When country-specific data were not available, regional data were used as a substitute ((8)). The decision to use regional data was based on the assumption that prevalence trends within specific geographic regions are often reflective of national trends. Each country was categorized as low (<2%) or intermediate-to-high (≥2%) ((9–11)), based on the pooled HBsAg prevalence ((8)). To capture changes over time (e.g., due to changes in hepatitis B vaccination policies in country-of-origin), immigration was stratified by time period. The time periods were based on Statistics Canada periods of immigration: ≤1990, 1991–2000, 2001–2010, and 2011–2021. The number of immigrants with CHB was estimated by multiplying the number of immigrants for each time period of immigration by the corresponding country or region-specific estimated prevalence for each respective time period (**Figure 1**). To account for uncertainty, plausible ranges were calculated by applying the same method to the lower and upper bounds of the estimated prevalence.

Both hepatitis C antibody prevalence (HCV antibodies), or history of HCV infection, and chronic hepatitis C (CHC) prevalence, defined as HCV-RNA positive were estimated. Countries were categorized as low (<2%) or intermediate-to-high (≥2%) ((12,13)) based on the country-specific prevalence of HCV antibodies estimated from the World Health Organization global hepatitis report ((14)) and region-specific estimates from Gower *et al*. ((15)), which were used as a substitute when country-specific estimates were not available. Chronic hepatitis C prevalence estimates were obtained from the Polaris Observatory ((7)). Since decade-specific data for CHC prevalence were not available, data were stratified as prior to 2016 and 2016–2021, accounting for the impact of the wide-scale implementation of direct-acting antivirals (DAA) curative treatment. The number of immigrants with a history of HCV infection and those with CHC were estimated by multiplying the number of immigrants from each period of immigration by the corresponding country or region-specific estimated prevalence for each respective time period (Figure 1). Again, plausible ranges were calculated by applying the same method to the lower and upper bounds of the estimated prevalence.

## Results

In 2021, there were an estimated 8,359,005 immigrants in Canada ((5)). An estimated 67% of immigrants originated from countries with CHB prevalence ≥2% (list of countries in **Box 1**). An estimated 15% originated from countries with CHC prevalence ≥2% (**Box 2**).

Box 1 **Countries with hepatitis B pooled seroprevalence (HbsAg positivity) ≥2%, sorted alphabetically**

**Table:** 

Afghanistan Albania Algeria American Samoa Angola Anguilla Antigua and Barbuda Aruba Azerbaijan Bahamas Bahrain Bangladesh Belarus Benin Bermuda Bhutan Bolivia Bonaire, Sint Eustatius and Saba Botswana Brunei Darussalam Bulgaria Burkina Faso Burundi Cabo Verde Cambodia Cameroon Cayman Islands Central African Republic Chad China Comoros Congo, Democratic Republic of the	Côte d'Ivoire Curaçao Cyprus Djibouti Dominica Dominican Republic Egypt Equatorial Guinea Eritrea Estonia Eswatini Ethiopia Fiji French Polynesia Gabon Gambia Georgia Ghana Grenada Guadeloupe Guam Guinea Guinea-Bissau Guyana Haiti Hong Kong India Indonesia Iran Italy Jamaica Jordan Kazakhstan	Kenya Kiribati Korea, North Korea, South Kuwait Kyrgyzstan Laos Lesotho Liberia Libya Lithuania Macao Madagascar Malawi Malaysia Maldives Mali Marshall Islands Martinique Mauritania Mauritius Micronesia, Federated States of Moldova Mongolia Montenegro Montserrat Mozambique Myanmar Namibia Nauru New Caledonia New Zealand	Niger Nigeria North Macedonia Northern Mariana Islands Oman Pakistan Papua New Guinea Philippines Puerto Rico Qatar Réunion Romania Russian Federation Rwanda Saint Helena, Ascension and Tristan da Cunha Saint Kitts and Nevis Saint Lucia Saint Martin Saint Vincent and the Grenadines Samoa Sao Tome and Principe Saudi Arabia Senegal Serbia Seychelles Sierra Leone Singapore Sint Maarten Solomon Islands Somalia South Africa, Republic of South Sudan	Sri Lanka Sudan Taiwan Tajikistan Tanzania Thailand Timor-Leste Togo Tonga Tunisia Turkey Turkmenistan Turks and Caicos Islands Uganda United Arab Emirates Uzbekistan Vanuatu Viet Nam Virgin Islands, British Virgin Islands, United States Yemen Zambia Zimbabwe

Source: Wong RJ, Brosgart CL, Welch S, Block T, Chen M, Cohen C, Kim WR, Kowdley KV, Lok AS, Tsai N, Ward J, Wong SS, Gish RG. An Updated Assessment of Chronic Hepatitis B Prevalence Among Foreign-Born Persons Living in the United States. Hepatology 2021;74 (2):607–26. https://doi.org/10.1002/hep.31782

Box 2 **Countries with hepatitis C seroprevalence (anti-HCV positivity) ≥2%, sorted alphabetically**

**Table:** 

Angola Armenia Azerbaijan Belarus Benin Burkina Faso Burundi Cabo Verde Cambodia Chad China, Province of Taiwan Congo Congo, Democratic Republic of the Côte d'Ivoire Egypt Equatorial Guinea Gabon Georgia Ghana Guinea Guinea-Bissau Italy Kazakhstan Kuwait Kyrgyzstan	Latvia Liberia Mali Mauritania Moldova Mongolia Niger Nigeria Pakistan Papua New Guinea Puerto Rico Romania Russian Federation Saint Helena, Ascension and Tristan da Cunha Sao Tome and Principe Senegal Sierra Leone Syrian Arab Republic Tajikistan Togo Turkmenistan Ukraine Uzbekistan West Bank and Gaza Strip

Sources: World Health Organization. Web Annex B. WHO estimates of the prevalence and incidence of hepatitis C virus infection by World Health Organization region, 2015. Global hepatitis report 2017. Geneva, CH: WHO; 2017. https://iris.who.int/bitstream/handle/10665/277005/WHO-CDS-HIV-18.46-eng.pdfGower E, Estes C, Blach S, Razavi-Shearer K, Razavi H. Global epidemiology and genotype distribution of the hepatitis C virus infection. J Hepatol 2014;61(1 Suppl):S45–57. https://doi.org/10.1016/j.jhep.2014.07.027

### Chronic hepatitis B virus prevalence

The estimated CHB prevalence among all Canadian immigrants was 4.03% (3.02%–5.08%) or 336,834 people (252,572–424,621), at the end of 2021. Among immigrants who came from intermediate-to-high-prevalence countries (≥2%), the estimated CHB prevalence was 5.57% (4.23%–6.96%), representing approximately 311,847 people (237,073–389,642). Whereas, among immigrants who came from low prevalence countries (<2%), the estimated CHB prevalence was 0.91% (0.56%–1.27%), or 24,988 people (15,500–34,979) (**Table 1**).

**Table 1:** Estimated prevalence of chronic hepatitis B virus (chronic hepatitis B) among immigrants in Canada, per time period of immigration and overall

Population	Population size estimate	≤1990		1991–2000		2001–2010		2011–2021		Overall	
		Prevalence (%)	Estimated number	Prevalence (%)	Estimated number	Prevalence (%)	Estimated number	Prevalence (%)	Estimated number	Prevalence (%)	Estimated number
Immigrants from countries with low prevalence (<2%)	2,759,465	0.84% (0.56%–1.14%)	10,791 (7,233–14,584)	1.14% (0.62%–1.69%)	4,457 (2,435–6,577)	0.93% (0.54%–1.36%)	4,415 (2,530–6,424)	0.87% (0.54%–1.21%)	5,324 (3,302–7,394)	0.91% (0.56%–1.27%)	24,988 (15,500–34,979)
Immigrants from countries with intermediate-to-high prevalence (≥2%)	5,599,485	7.01% (5.54%–8.50%)	82,718 (65,312–100,270)	6.77% (5.15%–8.44%)	75,885 (57,702–94,627)	5.53% (4.31%–6.78%)	80,631 (62,774–98,856)	3.94% (2.79%–5.21%)	72,612 (51,285–95,889)	5.57% (4.23%–6.96%)	311,847 (237,073–389,642)
All immigrants	8,358,950	3.80% (2.94%–4.66%)	93,510 (72,546–114,854)	5.32% (3.98%–6.70%)	80,341 (60,136–101,204)	4.41% (3.38%–5.45%)	85,046 (65,303–105,279)	3.18% (2.22%–4.21%)	77,936 (54,587–103,284)	4.03% (3.02%–5.08%)	336,884 (252,572–424,621)

Using time period of immigration, estimated CHB prevalence decreased for high prevalence countries, from 7.01% prior to 1990 to 3.94% between 2011–2021. Estimated CHB prevalence among immigrants from low-prevalence countries was relatively stable (0.84%–0.87%); however, the number of immigrants decreased from 10,791 prior to 1990 to 5,324 between 2011–2021 (Table 1).

**Figure 2:**
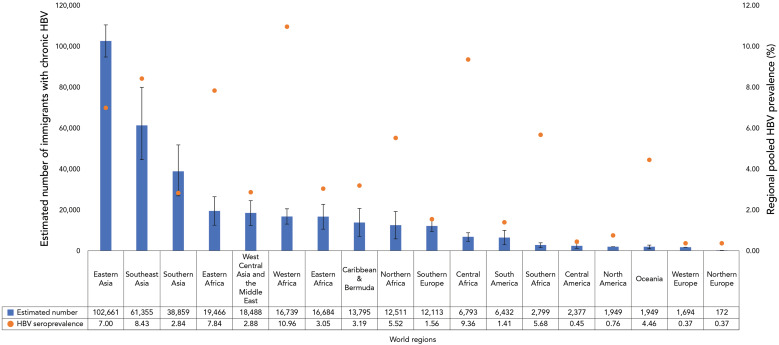
Estimated prevalence and number of immigrants in Canada with chronic hepatitis B, by world region, 2021 Abbreviation: HBV, hepatitis B virus

Although the pooled HBsAG prevalence rate was highest in Western Africa (10.96%) ((8)), the estimated number of immigrants to Canada with CHB from Western Africa was 16,739, representing only 4.9% of all estimated immigrants living with HBV. Alternatively, the highest estimated number of immigrants to Canada with CHB was from Eastern Asia (102,661), representing 30% of all estimated immigrants living with CHB, despite having a lower pooled HBsAG prevalence rate of 7.0% (**Figure 2**).

### Prevalence of hepatitis C virus antibodies

The estimated prevalence of HCV antibodies among all immigrants was 1.43% (0.91%–2.33%), or 119,432 people (76,216–194,635). Among immigrants who came from intermediate-to-high prevalence countries (≥2%), the estimated prevalence of HCV antibodies was 4.04%, compared to 0.96% among those who come from low prevalence countries (**Table 2**).

**Table 2:** Estimated prevalence of hepatitis C virus antibodies among immigrants in Canada, 2021

Population	Population size estimate	Prevalence (%)			Estimated number of immigrants with current or past HCV infection		
		Point estimate	Lower bound	Upper bound	Point estimate	Lower bound	Upper bound
Immigrants from countries with low prevalence (<2%)	7,082,480	0.96%	0.56%	1.54%	67,892	39,530	109,319
Immigrants from countries with intermediate-to-high prevalence (≥2%)	1,276,405	4.04%	2.87%	6.68%	51,540	36,686	85,316
All immigrants	8,358,885	1.43%	0.91%	2.33%	119,432	76,216	194,635

Abbreviation: HCV, hepatitis C virus

### Chronic hepatitis C virus prevalence

The estimated CHC prevalence among all immigrants was 0.78% (0.55%–1.31%), or 65,172 people (45,684–109,168) at the end of 2021. Among immigrants who came from intermediate-to-high-prevalence countries (≥2%), the estimated CHC prevalence was 2.20% (1.55%–3.49%), or 28,139 people (19,796–44,582). Among immigrants from countries with low CHC prevalence, estimated prevalence was 0.52% (0.37%–0.91%), or 37,032 people. When comparing by time period, the estimated CHC prevalence decreased slightly for the period of 2016–2021 (1.76%) compared to before 2016 (2.31%) (**Table 3**).

**Table 3:** Estimated prevalence of chronic hepatitis C virus among immigrants in Canada, per period of arrival and overall

Population	Population size estimate	Arrival before 2016		Arrival in 2016–2021		Overall	
		Prevalence (%)	Estimated number	Prevalence (%)	Estimated number	Prevalence (%)	Estimated number
Immigrants from countries with low prevalence (<2%)	7,082,480	0.53% (0.37%–0.91%)	31,680 (22,000–54,791)	0.49% (0.36%–0.91%)	5,352 (3,888–9,795)	0.52% (0.37%–0.91%)	37,032 (25,888–64,586)
Immigrants from countries with intermediate-to-high prevalence (≥2%)	1,276,405	2.31% (1.64%–3.53%)	23,838 (16,953–36,438)	1.76% (1.16%–3.32%)	4,301 (2,843–8,145)	2.20% (1.55%–3.49%)	28,139 (19,796–44,582)
All immigrants	8,358,885	0.79% (0.55%–1.30%)	55,519 (38,953–91,228)	0.73% (0.51%–1.35%)	9,653 (6,731–17,940)	0.78% (0.55%–1.31%)	65,172 (45,684–109,168)

**Figure 3:**
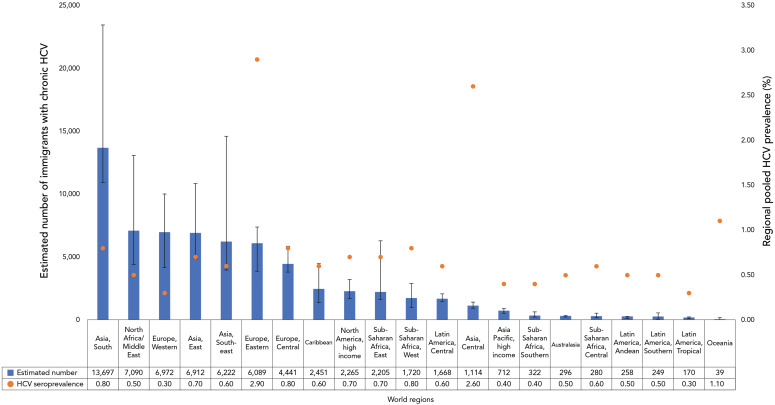
Estimated number of immigrants in Canada with chronic hepatitis C by world region, 2021 Abbreviation: HCV, hepatitis C virus

The regional CHC prevalence was highest in those from Eastern Europe (2.90%) ((7)). However, the highest estimated number of immigrants with CHC was from South Asia (13,697) representing 21% of all immigrants estimated to be living with CHC (**Figure 3**).

## Discussion

As far as is known, this is the first study to estimate the burden of both hepatitis B and C among immigrants at the national level in Canada. The results show that the prevalence of viral hepatitis among all immigrants (estimated at 4.03% for CHB, 1.43% HCV antibodies, and 0.78% for CHC) is higher than the latest published estimates for the general Canadian population (estimated at 0.68% for CHB, 0.99% HCV antibodies, and 0.56% for CHC) ((16)). However, when separated into immigrants from low- and intermediate-to-high-prevalence countries, results show that the prevalence of CHB, HCV antibodies, and CHC among immigrants from low-prevalence countries (<2%) (0.91%, 0.96% and 0.52%, respectively) is similar to the Canadian general population. The estimated prevalence among immigrants from intermediate-to-high-prevalence countries from this study was 5.57% for CHB, 4.04% for HCV antibodies, and 2.20% for CHC. This demonstrates that grouping all immigrants into one category masks important variation, and potentially over-estimates the burden of hepatitis B and C among immigrants. In addition, the estimated number of immigrants with CHB and CHC varied over time. This could be the result of changes in immigration patterns and policies, the implementation of HBV immunization, and the introduction of direct-acting antivirals for the treatment of HCV. Strengthening our understanding of the variation in hepatitis prevalence among immigrants can improve our ability to connect those in need to hepatitis B care services and hepatitis C curative treatment, enabling the development of targeted programming for those populations. Surveillance systems and research provide important insights into where action is needed, helping to tailor interventions and reduce the health impact of STBBIs in key populations.

Although there is limited national data for comparison, a study by Wong *et al*. estimated the prevalence of CHB among all Canadian immigrants in 2006 to be 4.81%. While this estimate falls within the plausible range of our estimate of 4.03% (3.02%–5.08%), it suggests a slight decrease in prevalence in recent years. Smaller-scale studies have also been conducted in Canadian provinces. A population-based study by Yasseen *et al*. ((11)) estimated the prevalence of CHB among immigrants from intermediate-to-high-prevalence countries living in Ontario at 5.4%, which aligns with this study’s estimate of 5.57% among this group. While comparable national estimates for HCV are not available, modelling studies estimating the prevalence of HCV have been conducted at the provincial level. A study by Forouzannia *et al*. ((17)) estimated a CHC prevalence of 2.0% among all immigrants in Québec in 2016, and Yasseen *et al*. ((18)) estimated an HCV antibody prevalence of 0.7% among immigrants in Ontario in 2014. Although both of these estimates differ from our national estimates of 0.78% CHC prevalence and 1.43% HCV antibody prevalence, it may be indicative of regional variability in immigration across Canada.

### Limitations

The methods used in our study present limitations. First, the use of country-of-origin prevalence data to estimate the burden of CHB and CHC among immigrants living in Canada may lead to overestimates. This phenomenon, known as the healthy immigrant effect, suggests that individuals who immigrate may differ from those who remain in their country of origin in terms of age structure, risk profile, socioeconomic status and, ultimately, health status ((19,20)). Nonetheless, while the true burden may more closely align with the lower bounds of our estimate, even a conservative interpretation of these estimates indicates a disproportionate disease burden among immigrants to Canada. Second, although the method of applying country-of-origin specific prevalence has been found to be a good proxy for the expected prevalence in the immigrant population ((13)), this method also brings inherent uncertainty and could lead to either an over or underestimation of the prevalence among immigrants in Canada. Uncertainty in this method arises from the reliance on a smaller pool of studies for period-specific prevalence estimates, and a lack of prevalence data from smaller countries used in global hepatitis prevalence reports. This can lead to an over-reliance on regional data in some cases, and a bias towards larger countries due to weighted averages being more heavily influenced by countries with larger populations. Furthermore, the data extracted for this study was from published data, which was aggregated data. Thus, potential confounders or effect modifiers cannot be addressed. Third, while time-period-specific immigration and prevalence was considered to increase estimation accuracy, countries initially categorized as low (<2%) or intermediate-to-high (≥2%) may change categories over time and may lead to an under- or over-estimation of prevalence. Fourth, prevalence estimates included in published global studies were selected based on how well their results could be extrapolated to a country’s general population, and in the study used for CHB prevalence, certain groups known to be at high risk for hepatitis B infection were excluded. Estimates included in this study should therefore be interpreted within the context of their plausible ranges because of these factors. Fifth, the estimates are national level only and have not been broken down by province/territory. The countries of origin of immigrants living in each province/territory varies and can be driven by linguistic preferences, cultural links, and job availability. Therefore, national-level estimates may not be helpful to support regional-specific programs tailored for immigrants. Lastly, the analysis does not account for differences by age and gender, which are important considerations to understanding the population at risk, and would help inform programming for specific subgroups within the immigrant population.

## Conclusion

The availability of safe and effective hepatitis B vaccines, along with antiviral treatment capable of preventing transmission ((21,22)), and the ability to effectively cure hepatitis C, have created conditions in which the elimination of hepatitis B and C is increasingly within reach. However, while the prevalence of viral hepatitis within the general population of Canada is relatively low, some immigrants experience a higher burden of disease due to potential exposure in their countries of origin. This demographic factor brings additional challenges in achieving the goal of elimination. These data are an important first step in describing the burden of viral hepatitis among immigrants. Additional data on the prevalence of hepatitis B and C among immigrants in Canada, as well as region, age, and gender specific data, are needed to help address the specific needs of immigrant populations and improve health outcomes for those most affected.
